# Effect of Kisspeptin on the Developmental Competence and Early Transcript Expression in Porcine Oocytes Parthenogenetically Activated with Different Methods

**DOI:** 10.1155/2018/3693602

**Published:** 2018-02-21

**Authors:** Islam M. Saadeldin, Ayman Abdel-Aziz Swelum, Aaser M. Abdelazim, Essam A. Almadaly

**Affiliations:** ^1^Department of Animal Production, College of Food and Agricultural Sciences, King Saud University, Riyadh 11451, Saudi Arabia; ^2^Department of Physiology, Faculty of Veterinary Medicine, Zagazig University, Zagazig 44519, Egypt; ^3^Department of Theriogenology, Faculty of Veterinary Medicine, Zagazig University, Zagazig 44519, Egypt; ^4^Department of Biochemistry, Faculty of Veterinary Medicine, Zagazig University, Zagazig 44519, Egypt; ^5^Department of Basic Medical Sciences, College of Applied Medical Sciences, University of Bisha, Bisha, Saudi Arabia; ^6^Department of Theriogenology, Faculty of Veterinary Medicine, Kafrelsheikh University, El-Geish Street, Kafrelsheikh 33516, Egypt

## Abstract

Recent studies showed the modulatory effect of kisspeptin (KP) on calcium waves through the cell membrane and inside the cell. Spermatozoon can induce similar ooplasmic calcium oscillations at fertilization to trigger meiosis II. Here, we evaluated the effect of KP supplementation with 6-dimethylaminopurine (6-DMAP) for 4 h on embryonic development after oocyte activation with single electric pulse, 5 *µ*M ionomycin, or 8% ethanol. Compared to control nonsupplemented groups, KP significantly improved embryo developmental competence electric- and ethanol-activated oocytes in terms of cleavage (75.3% and 58.6% versus 64% and 48%, respectively, *p* < 0.05) and blastocyst development (31.3% and 10% versus 19.3% and 4%, respectively, *p* < 0.05).* MOS* expression was increased in electrically activated oocytes in presence of KP while it significantly reduced* CCNB1* expression. In ionomycin treated group, both* MOS* and* CCNB1* showed significant increase with no difference between KP and control groups. In ethanol-treated group, KP significantly reduced* CCNB1* but no effect was observed on* MOS* expression. The early alterations in* MOS* and* CCNB1* mRNA transcripts caused by KP may explain the significant differences in the developmental competence between the experimental groups. Kisspeptin supplementation may be adopted in protocols for porcine oocyte activation through electric current and ethanol to improve embryonic developmental competence.

## 1. Introduction

In mammals, meiosis II resumes in mature oocytes after fertilization and is mainly mediated by the highly repetitive calcium (Ca^2+^) signals that last for 2-3 h rather than a single Ca^2+^ rise [1, 2]. Ca^2+^ modulates the transition of the cell from one meiotic phase and cell cycle control checkpoints to the following phase till the extrusion of the second polar body [3]. Lack of intracellular Ca^2+^ elevation prevents spontaneous meiosis resumption in vitro [4]. Moreover, injection of Ca^2+^ in mouse oocytes has been shown to cause parthenogenetic activation and subsequent embryonic development [1].

The c-Mos* (MOS)* proto-oncogene is a serine/threonine kinase expressed in vertebrate oocytes and plays a crucial role in meiosis and germ cell development [5, 6].* MOS* is functionally expressed during G2/M progression of oocyte cell cycle [7] and was found to be expressed highly in oocytes while it reduced with the cleavage and mitosis of the zygote [8].* MOS* activates mitogen-activated protein kinase (MAPK), which in turn activates M-phase promoting factor (MPF) [6]. MPF is a protein complex comprised of a catalytic subunit p34^cdc2^ with serine-threonine kinase activity and a regulatory subunit cyclin B* (CCNB1)*. MPF is regulated by the binding of* CCNB1* with cyclin-dependent kinase 1 (Cdc2) and phosphorylation of threonine 161 and dephosphorylation of tyrosine 15 and threonine 14 [9]. Notably, there is a regulatory interplay between Ca^+2^,* MOS*, and* CCNB1* in a feedback manner to regulate the exit from meiosis II [10–15].

Parthenogenetic activation of porcine oocytes has been carried out using different methods such as electrical stimulus, chemical activation, and ethanol treatment [16–19]; however, the underlying molecular mechanism is questionable. During parthenogenesis protocols, activation is usually followed by the inhibition of protein phosphorylation with 6-dimethylaminopurine (6-DMAP), a protein kinase inhibitor, resulting in efficient oocyte activation and development of parthenotes into the blastocyst stage [20]. 6-DMAP inhibits MPF reactivation, thereby triggering a kinetic similar to that occurring after fertilization [21, 22]. However, interfering with one or several kinases involved in other cellular functions may alter the subsequent cellular events.

Interestingly, cyclin degradation is activated by fertilization; however this activation is transitory since cyclin B1 levels recover between Ca^2+^ oscillation spikes. Therefore, continued cyclin degradation at basal Ca^2+^ levels requires multiple spikes to complete meiosis II [23].

Kisspeptin (KP) is a multifunctional peptide known for its function in reproductive endocrinology, cancer, cardiovascular system, and urogenital system [24, 25]. Our previous report and other studies showed that kisspeptin receptor* (KISS1R)* is functional throughout the oocyte maturation until early embryonic stages [26–31]. In addition, KP supplementation during in vitro maturation (IVM) increases the maternal mRNA transcripts, including that for* MOS* [31]. KP is thought to modulate Ca^2+^ oscillations through the cell membrane and inside the cell, including spermatozoa [32–38]. Activation of KISS1R results in a biphasic rise in intracellular Ca^2+^ characterized by a short and transient phase followed by a sustained phase [37].

Therefore, this study aimed to examine whether KP, as a Ca^2+^ modulator, can differentially support the transition of oocytes from MII stage to further embryonic developmental stages when oocytes activated with electric stimulus, ionomycin, or ethanol through investigating the effect of KP supplementation on the expression of early maternal transcripts involved in meiotic progression,* MOS* and* CCNB1*.

## 2. Materials and Methods

### 2.1. Chemicals

Kisspeptin (112–121) amide was obtained from Phoenix Pharmaceuticals Inc. (Burlingame, CA, USA). Other chemicals were obtained from Sigma-Aldrich Corp. (St. Louis, MO, USA), unless otherwise stated.

### 2.2. Collection of Cumulus-Oocyte Complexes (COCs) and Their In Vitro Maturation

Ovaries obtained from a local abattoir were transported to the laboratory within 4 h in 0.9% (v/v) sodium chloride (NaCl) solution at 30–33°C. Follicular contents from antral follicles, 2–6 mm in diameter, were collected using an 18-gauge needle attached to a 10 mL sterile syringe. COCs enclosed by more than three layers of compact cumulus cells and with evenly granulated cytoplasm were selected and washed thrice with HEPES-buffered tissue culture medium-199 (TCM-199) supplemented with 2 mM sodium bicarbonate (NaHCO_3_), 5 mg/mL bovine serum albumin (BSA), and 1 *μ*L/mL gentamycin sulfate (Caisson Lab. Inc., Smithfield, UT, USA). Groups of 50 COCs were cultured in four-well dishes (Falcon, Becton Dickinson Ltd., Plymouth, UK) in 500 *μ*L maturation medium comprising bicarbonate-buffered TCM-199 supplemented with 10% (v/v) porcine follicular fluid, 10 *μ*g/mL follicle-stimulating hormone (FSH), 1 *μ*g/mL luteinizing hormone (LH), 1 *μ*g/mL 17*β*-estradiol, 20 ng/mL epidermal growth factor (EGF), 1 *μ*L/mL insulin-transferrin-selenium (ITS), 0.3 *μ*M cysteamine, 0.15 mg/mL L-glutamine, and 1 *μ*L/mL gentamycin sulfate at 39°C in a humidified atmosphere of 5% CO_2_ for 44 h (two stages, 22 h each with change of medium for the second stage).

### 2.3. Parthenogenetic Activation and KP Supplementation

After 44 h of IVM, oocytes and cumulus cells were separated by pipetting with 0.1% hyaluronidase in Dulbecco's phosphate-buffered saline (PBS) supplemented with 0.1% polyvinyl alcohol. Denuded oocytes were randomly separated into three groups and subjected to one of the following parthenogenetic activation methods: (1) in electrical activation, oocytes were activated with a single DC pulse of 1.5 kV/cm for 100 *μ*s in mannitol (0.25 M) using BTX Electro Cell Manipulator 2001 designed electrodes chamber (BTX, Inc., San Diego, CA, USA); (2) in ionomycin activation, oocytes were incubated in TCM-199 supplemented with 10% fetal bovine serum (FBS) and 5 *μ*M ionomycin for 5 min in a dark chamber; or (3) in ethanol activation, oocytes were incubated in TCM-199 containing 10% FBS and 8% ethanol for 10 min. Activated oocytes were washed in TCM and cultured in 500 *μ*L of 4 mM 6-DMAP with or without 1 *μ*M KP for 4 h at 39°C in an atmosphere of 5% CO_2_, 5% O_2_, and 90% N_2_ to produce diploid zygotes.

### 2.4. Experimental Design

Denuded oocytes were randomly allocated into three groups, 75 oocytes each, and incubated in 50 *μ*L microdrops of TCM-199 supplemented with 10% fetal bovine serum (FBS) for 10 min. Oocytes were then activated with specific treatment (electric, ionomycin, or ethanol activation), and then, a pool of oocytes (*n* = 25) was immediately collected from each group to extract mRNA (0 h after activation). After incubation with 6-DMAP with or without KP, another pool of oocytes (*n* = 25) was collected to extract mRNA from each group (4 h after activation batch). The remaining 25 oocytes were then used for in vitro culture. The experiment was repeated six times and the data were presented as mean ± SEM.

### 2.5. In Vitro Culture of Embryos

Presumptive zygotes were washed and cultured in 30 *μ*L microdrops of porcine zygote medium-5 covered with mineral oil at 39°C in presence of 5% CO_2_, 5% O_2_, and 90% N_2_. Embryos were evaluated for cleavage and blastocyst formation on days 2 and 7, respectively.

### 2.6. Real-Time qPCR for Relative Quantification of mRNA Transcripts

Cumulus-free oocytes (*n* = 25 for each replicate, confirmed by visual examination of each individual oocyte) at different stages of activation were collected, washed twice with PBS, and stored at −80°C in diethylpyrocarbonate- (DEPC-) treated water until analysis. Total RNAs were extracted from all samples following the manufacturer's protocol (iNtRON Biotechnology, Seoul, Korea). RNA concentration and purity were evaluated using a NanoDrop 2000 spectrophotometer (Thermo Fisher Scientific, Waltham, MA, USA) by calculating the ratios of absorbance at 260 and 280 nm; samples showing values ≥1.8 for *A*260/*A*280 were used for reverse transcription (RT). RT was carried out at 50°C for 50 min. Individual RT reaction was performed using random hexamer, 100 ng of total RNA and superscript III reverse transcriptase (Invitrogen, Carlsbad, CA, USA) in a 20-*μ*L reaction volume. Complementary DNA (cDNA) concentration was measured through NanoDrop spectrophotometer. Relative quantitative real-time polymerase chain reaction (qPCR) was carried out according to the Takara Bio Inc. guidelines. A total of 22 *μ*L PCR reaction was made by adding 0.1 *μ*g cDNA, 1 *μ*M forward primer, 1 *μ*M reverse primer, 8 *μ*L SYBR Premix Ex Taq, 0.4 *μ*L ROX reference (Takara Bio Inc. Shiga, Japan), and 9.6 *μ*L nuclease-free water (Ambion Inc., Austin, TX, USA). The reaction was performed on 7300 Real-Time PCR System (Applied Biosystems, Forest City, CA, USA) according to the company's instructions using the following program: 95°C for 10 min, followed by 40 cycles of 95°C for 10 s, 60°C for 20 s, and 72°C for 40 s.* MOS* and* CCNB1* mRNA transcripts were relatively quantified, with gene for glyceraldehyde 3-phosphate dehydrogenase* (GAPDH)* used as a normalization control [39] using 2^−ΔΔCt^ method [ΔCt Experimental = Ct (test gene) − Ct* (GAPDH)*; ΔCt Control = Ct (test gene) − Ct* (GAPDH)*; and ΔΔCt = ΔCt Experimental −  ΔCt Control] [40]. Primer sequences, annealing temperatures, and approximate sizes of the amplified fragments are listed in Table 1. To determine primers specificity, melting curves for each primer pair were evaluated by Applied Biosystems 7300 Real-Time PCR apparatus-associated software and the product size was confirmed by gel electrophoresis of PCR products on agarose 1.5% referred to by 1 Kb DNA ladder. Samples from four replicates and three technical replicates were recorded for each qPCR sample reaction. Data were recorded and used for statistical analysis and presented as mean ± SEM.

### 2.7. Statistical Analysis

The COCs and oocytes were randomly distributed within each experimental group and experiments were repeated at least five times. Embryo development proportions were calculated for each replicate and relative quantification of transcripts was analyzed by one-way ANOVA using SAS (SAS Institute, Cary, NC, USA, 2000) which was followed by Tukey's Multiple Range Test to determine the differences among the groups. Values were presented as means ± standard error of the mean (SEM). Data were considered statistically significant at value of *p* < 0.05.

## 3. Results and Discussion

**Figure 1:**
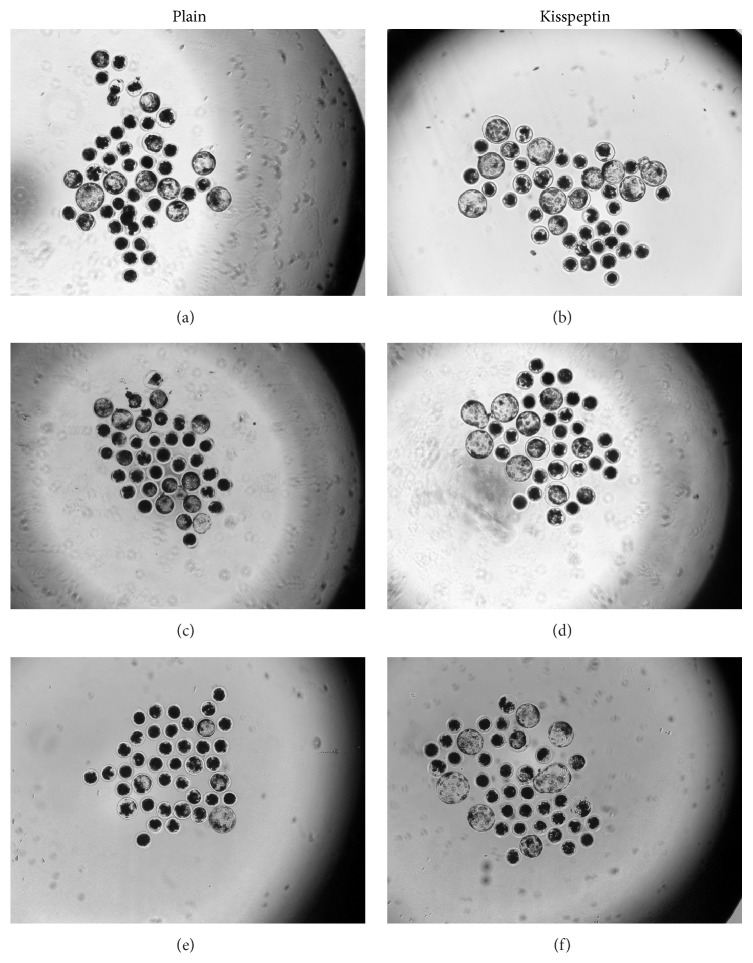
Preimplantation embryonic development of porcine oocytes after parthenogenetic activation with electrical stimulation, ionomycin, and ethanol. (a), (c), and (e) development without KP (plain), and (b), (d), and (f) with KP supplementation during incubation with 6-DMAP.

Results showed that supplementation of 6-DMAP culture with KP (KP+) significantly increased embryo cleavage proportion in oocytes activated with electric pulse and ethanol (75.42 ± 2.9% and 58.0 ± 0.6%, respectively) as compared with the control groups (KP−) (Table 2) with maximum effect observed with electrical activation (*p* < 0.05). On the other hand, KP supplementation had no advantageous effect on the oocytes activated with ionomycin (Table 2, Figure 1). Similar trend was observed for blastocysts; KP-supplemented groups activated with electric pulse and ethanol showed a significant increase in blastocyst proportion (31.75 ± 0.9% and 10.25 ± 0.25%, respectively) as compared with control groups (Table 2, *p* < 0.05). On the other hand, KP supplementation showed no effect on blastocyst formation in oocytes activated with ionomycin.

Additionally, a significant increase in embryo cleavage and blastocyst was observed following oocyte activation with electric pulse (64.25 ± 2.1% and 19.5 ± 0.3%, respectively) and ionomycin (63.25 ± 0.25% and 22.25 ± 0.8%, respectively) as compared to that in ethanol-activated oocytes (47.5 ± 0.3% and 4.0 ± 0.6%, respectively, *p* < 0.05) (Table 2, Figure 1). This is consistent with the results by Cheng et al. [16], wherein the electrical and ionomycin activation resulted in a threefold increase in developmental competence of porcine oocytes as compared with the ethanol-mediated activation. Moreover, ethanol treatment in oocytes was reported to yield a very low percentage of cleavage and blastocyst formation [19]. On the contrary, Koo et al. [41] showed that electrical activation significantly increased the cleavage and blastocyst development as compared with ionomycin activation.

Recent reports have described the mechanism of action of KP through the modulation of Ca^2+^ oscillation in different cell types, including spermatozoa [32–36]. In addition, KP is known to cause biphasic increase in intracellular Ca^2+^ characterized by a short and transient phase followed by a sustained phase [37]. Following oocyte activation, the oscillation in Ca^2+^ signals, in particular the early Ca^2+^/calmodulin-dependent kinase pathways, results in the emission of the second polar body [42]. The recruitment of maternal mRNAs is thought to be a graded response to the number of Ca^2+^ oscillations experienced by the oocyte [43]. In addition, artificial methods of oocyte activation may result in the recruitment of the maternal mRNA in the absence of Ca^2+^ elevation [44], indicative of the indirect effect of Ca^2+^ on the process downstream of cell cycle resumption. Electric pulse showed a higher and longer Ca^2+^ transient in the oocyte immediately after its activation [45]. Moreover, electrical activation induces a rapid reduction in histone H1 kinase activity [46]. However, the elevation in intracellular levels of free Ca^2+^ by ionomycin was less effective for parthenogenetic activation of oocytes than that caused by electrical pulse [47].

Studies have reviewed the different mechanistic pathways for porcine parthenogenesis [48, 49]. In particular, electrical pulse causes influx of Ca^2+^ by increasing the permeability of the oolemma [50, 51], while 7% ethanol and calcium ionophores cause a surge in the intracellular Ca^2+^ from Ca^2+^ stores by increasing the intracellular pH [47, 52–54]. The sodium-hydrogen (Na^+^/H^+^) antiporter and the bicarbonate-chloride (HCO_3_^−^/Cl^−^) exchanger are suggested to play no role during calcium ionophore treatment; however, ethanol treatment in oocytes may be dependent upon either Na^+^ or HCO_3_^−^ flux (or both) [53]. On the other hand, calcium ionophores inhibit the vacuolar-type H^+^ ATPases, thereby increasing the intracellular pH [53]. The pH rise plays an important role in activating oocyte metabolism and increasing DNA and protein synthesis [55]. Since the Ca^+2^ ions have two opposing roles during Xenopus oocyte maturation, it negatively regulates meiosis entry by delaying the activation of the cell cycle machinery (downstream of protein kinase A and upstream of MOS), while on the other hand, it is required for completion of meiosis [56]. Therefore, modulation of temporal calcium influx to the ooplasm is essential for successful meiosis. The three activation methods use calcium signals as the main pathway for oocyte activation; however, how acute or repetitive calcium influx is and for how long this influx continues are questions for clarifying the difference between these methods for supporting meiosis II and early embryo development.

We investigated the possible cause of the difference between activation methods with or without KP supplementation.* MOS* and* CCNB1* transcripts were relatively quantified through real-time PCR immediately after activation (0 h after activation), and 4 h after activation and culture in 6-DMAP (4 h after activation) with or without KP. Statistical analysis showed significant interaction among the treatments and the temporal expression of* MOS* and* CCNB1*.

It has been clarified that 6-DMAP, a phosphatase inhibitor, caused a higher and longer Ca^2+^ transient and may activate oocytes by changing Ca^2+^ oscillations in oocytes and inhibiting phosphotyrosine dephosphorylation after parthenogenetic activation [45]. However, oocytes activated with calcium ionophores and 6-DMAP displayed abnormal pattern of karyokinesis during the first cell cycle [57]. Although 6-DMAP-mediated inhibition of protein kinase is an efficient way to induce oocyte activation, interfering with one or several kinases involved in other functions may be deleterious for cellular events after activation. Therefore, we aimed to examine whether KP may act as a Ca^2+^ modulator during this period.

**Figure 2:**
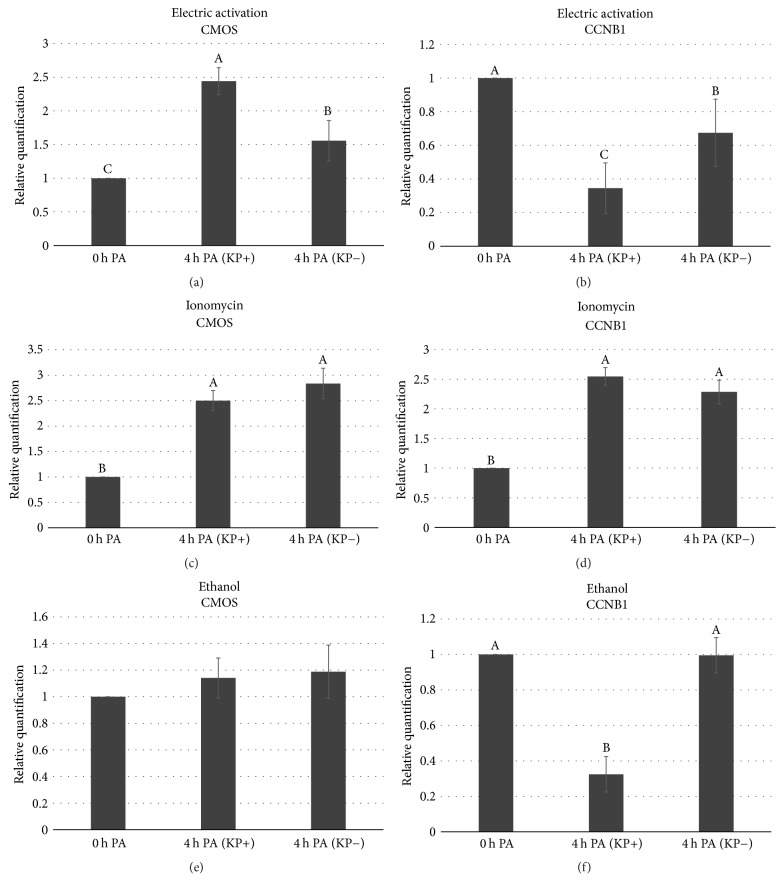
Effect of kisspeptin supplementation after porcine oocyte activation (with electric activation, ionomycin, and ethanol) on temporal expression of* MOS* and* CCNB1* mRNAs during culture in 6-dimethylaminopurine (6-DMAP) by real-time PCR. The relative gene abundance was normalized to* GAPDH *levels. The mRNA expression in 0 h after activation (0 h PA) was arbitrarily set as onefold. Data are presented as mean ± SEM. Significant difference (*p* < 0.05) is indicated by different letters A, B, and C.

Kisspeptin supplementation after 4 h from activation resulted in differential alteration in the expression patterns of* MOS* and* CCNB1* in different treatment groups; it significantly increased* MOS* expression (2.5-fold, Table 2) when compared with both control (KP−) and 0 h after activation in electrically activated oocytes (*p* < 0.05; Figures 2(a) and 2(b)). In ethanol-treated group, KP significantly decreased the level of* CCNB1* by 0.3-fold when compared with 0 h after activation and control nonsupplemented group (*p* < 0.05); however, no effect was observed on* MOS* (Figures 2(e) and 2(f)). On the other hand, oocytes treated with ionomycin showed no difference on* MOS* and* CCNB1* both with and without KP supplementation (*p* = 0.9), although* MOS* and* CCNB1* expressions significantly increased (~2.5-fold) after 4 h from activation (Figures 2(c) and 2(d), *p* < 0.05).

Some reports showed that mRNA expression of proto-oncogenes (such as* MYC*) is tightly and coordinately regulated and rapidly turned over with half-lives of 9–40 min [23, 58, 59]. Moreover, transcription rates for transiently expressed genes and mRNA stability are often rapidly and coordinately affected by calcium-dependent signaling pathways [60, 61]. These observations might explain the rapid transcriptional changes in proto-oncogene* MOS* and* CCNB1* associated with oocyte stimulation and its incubation with calcium modulator, KP.

The early changes in cyclin B and c-Mos complexes are critical for pronuclear formation; after oocyte fertilization or parthenogenetic activation, an increase in intracellular Ca^2+^ causes degradation of c-Mos protein, inactivation of cytostatic factor (CSF) and MPF, and release of eggs from meiotic metaphase arrest [62]. These results suggest that the early changes in* MOS* and* CCNB1* may influence the embryonic developmental competence, including cleavage and subsequent mitosis stages until the blastocyst formation. On the other hand, Lazar et al. [63] clarified that* MOS* mRNA expression is associated with meiosis progression and occurs independent of MAPK. In addition, Koo et al. [41] showed inactivation of MPF and MAPK after oocyte activation with single electrical stimulus.

In the current study, KP increased the level of* MOS* (2.5-fold) in electrically activated oocytes, highlighting the need of* MOS* for successful completion of cell cycle progression after activation [6, 7]. Moreover, supplementation of the electrically or ethanol-activated oocytes with KP significantly reduced the level of* CCNB1* transcript, which is essential for MPF inhibition to allow meiosis II to proceed [9]. Interestingly, the interplay between* MOS* and* CCNB1* plays an important role during meiosis in mammalian oocytes [10–15]. Physiologically, a spermatozoa Ca^2+^ signal induces degradation of cyclin B1 which in turn results in exit of oocyte from metaphase II arrest and permits extruding the second polar body [14]. On the other hand,* MOS* is critical for protracted metaphase II arrest, but it is not required for its establishment [64, 65]. Paradoxically, MPF activity may negatively regulate the MOS pathway; Yamamoto et al. [66] showed that when MPF activity reaches a critical lower level, the c-Mos/MAPK pathway suppresses cyclin B degradation in order to elevate MPF levels, while elevation of MPF beyond a critical upper level activates cyclin B degradation.

## 4. Conclusion

Kisspeptin supplementation with 6-DMAP culture improved the developmental competence of oocytes activated with electric pulse and 8% ethanol. Relative quantitative analysis of the transcript showed changes associated with the efficient activation of oocytes;* MOS* expression was increased after electric and ionomycin activation and this effect was magnified in presence of KP in electrically activated oocytes. This could explain the difference in cleavage and blastocyst development observed in the two groups as compared with the ethanol-activated group. On the other hand,* CCNB1* expression was decreased in electric and ethanol-activated oocytes treated with KP. This result might also explain the interplay between different regulatory components of calcium ions, MOS/CCNB1/MAPK pathway to exit from oocyte metaphase II arrest which in turn reflects difference in embryo cleavage, and blastocyst development observed between groups treated with the calcium modulatory peptide KP.

We suggest that KP supplementation may be adopted in the protocol for electric and ethanol activation of porcine oocyte to improve the embryonic developmental competence.

## Figures and Tables

**Table 1:** Primer sequences and product size used for real-time quantitative PCR.

Gene	Forward 3′→5′	Reverse 3′→5′	*T*_*m*_ (°C)	Product size (bp)	Information
*MOS*	GGGAGCAACTGAACTTGGAG	AGAATGTTCGCTGGCTTCAG	60	115	NM_001113219 (accession number)
*CCNB1*	CAACTGGTTGGTGTCACTGC	TTCCATCTGCCTGATTTGGT	60	126	NM_001170768 (accession number)
*GAPDH*	ACACTCACTCTTCTACCTTTG	CAAATTCATTGTCGTACCAG	60	90	DQ845173.1 (GenBank)

**Table 2:** Embryonic development after activation of oocytes with electric pulse, ionomycin, and ethanol with or without supplementation of kisspeptin during 6-DMAP culture.

	Electric		Ionomycin		Ethanol	
	KP+	KP−	KP+	KP−	KP+	KP−
Oocyte Number^*∗*^	150	150	150	150	150	150
Cleavage%	5.42 ± 2.9^a^	64.25 ± 2.1^b^	63.25 ± 2.4^b^	3.25 ± 0.25^b^	58.0 ± 0.6^b^	47.5 ± 0.3^c^
Blastocyst%	31.75 ± 0.9^a^	19.5 ± 0.3^b^	22.0 ± 0.6^b^	22.25 ± 0.8^b^	10.25 ± 0.25^c^	4.0 ± 0.6^d^
Fold change of MOS^$^	2.5	1.61	2.5	2.8	1.1	1.18
Fold change of CCNB1^$^	0.4	0.65	2.5	2.25	0.3	0.98

^*∗*^6 replicates with average 25 oocytes per each replicate. The proportion of cleavage and blastocyst was calculated for each replicate and data expressed as mean ± SEM. ^$^The mean of fold change in *MOS* and *CCNB1* expression in oocytes at 0 h and 4 h after activation = expression level at 4 h divided by the expression at 0 h (values are shown in Figure 2). ^a,b,c,d^Values carrying different superscripts are considered significant when *p* < 0.05.

## Abbreviations

6-DMAP: 6-Dimethylaminopurine
MOS: c-MOS or proto-oncogene serine/threonine-protein kinase Mos
CCNB1: G2/mitotic-specific cyclin-B1
MPF: Mitosis-promoting factor or M-Phase-promoting factor
MAPK: Mitogen-activated protein kinase
KP: Kisspeptin
KISS1R: Kisspeptin receptor or KiSS1-derived peptide receptor or GPR54.

## Additional Points

*Availability of Data and Material.* All data generated or analyzed during this study are included in this article. Any inquiries should be sent directed to the corresponding author.

## References

[1] Jones K. T., Carroll J., Whittingham D. G. (1995). Ionomycin, thapsigargin, ryanodine, and sperm induced Ca^2+^ release increase during meiotic maturation of mouse oocytes. *The Journal of Biological Chemistry*.

[2] Stricker S. A. (1999). Comparative biology of calcium signaling during fertilization and egg activation in animals. *Developmental Biology*.

[3] Whitaker M., Patel R. (1990). Calcium and cell cycle contro. *Development*.

[4] Carroll J., Jones K. T., Whittingham D. G. (1996). Ca^2+^ release and the development of Ca^2+^ release mechanisms during oocyte maturation: a prelude to fertilization. *Reviews of Reproduction*.

[5] Hashiba Y., Asada Y., Heikinheimo O., Lanzendorf S. E., Mizutani S. (2001). Microinjection of antisense c-mos oligonucleotides prevents the progression of meiosis in human and hamster oocytes. *Fertility and Sterility*.

[6] Sagata N. (1997). What does Mos do in oocytes and somatic cells?. *BioEssays*.

[7] Ferrell J. E. (1999). Xenopus oocyte maturation: new lessons from a good egg. *Bioessays*.

[8] Heikinheimo O., Lanzendorf S., Baka S., Gibbons W. (1995). Cell proliferation and apoptosis: cell cycle genes c-mos and cyclin-B1 are expressed in a specific pattern in human oocytes and preimplantation embryos. *Human Reproduction*.

[9] Wehrend A., Meinecke B. (2001). Kinetics of meiotic progression, M-phase promoting factor (MPF) and mitogen-activated protein kinase (MAP kinase) activities during in vitro maturation of porcine and bovine oocytes: species specific differences in the length of the meiotic stages. *Animal Reproduction Science*.

[10] Castro A., Peter M., Magnaghi-Jaulin L. (2001). Cyclin B/cdc2 induces c-Mos stability by direct phosphorylation in *Xenopu*s oocytes. *Molecular Biology of the Cell (MBoC)*.

[11] Frank-Vaillant M., Haccard O., Ozon R., Jessus C. (2001). Interplay between Cdc2 kinase and the c-Mos/MAPK pathway between metaphase I and metaphase II in Xenopus oocytes. *Developmental Biology*.

[12] Haccard O., Jessus C. (2006). Redundant pathways for Cdc2 activation in Xenopus oocyte: either cyclin B or Mos synthesis. *EMBO Reports*.

[13] Ito J., Shimada M., Hochi S., Hirabayashi M. (2007). Involvement of Ca^2+^-dependent proteasome in the degradation of both cyclin B1 and Mos during spontaneous activation of matured rat oocytes. *Theriogenology*.

[14] Madgwick S., Jones K. T. (2007). How eggs arrest at metaphase II: MPF stabilisation plus APC/C inhibition equals cytostatic factor. *Cell Division*.

[15] Zhang D.-X., Cui X.-S., Kim N.-H. (2010). Molecular characterization and polyadenylation-regulated expression of cyclin B1 and Cdc2 in porcine oocytes and early parthenotes. *Molecular Reproduction and Development*.

[16] Cheng W.-M., Sun X.-L., An L. (2007). Effect of different parthenogenetic activation methods on the developmental competence of in vitro matured porcine oocytes. *Animal Biotechnology*.

[17] Liu S., Cui K., Li H. L. (2015). Comparison of chemical, electrical, and combined activation methods for in vitro matured porcine oocytes. *In Vitro Cellular & Developmental Biology—Animal*.

[18] Roh S., Hwang W.-S. (2002). In vitro development of porcine parthenogenetic and cloned embryos: comparison of oocyte-activating techniques, various culture systems and nuclear transfer methods. *Reproduction, Fertility and Development*.

[19] Yi Y. J., Park C. S. (2005). Parthenogenetic development of porcine oocytes treated by ethanol, cycloheximide, cytochalasin B and 6-dimethylaminopurine. *Animal Reproduction Science*.

[20] Paffoni A., Brevini T. A. L., Somigliana E., Restelli L., Gandolfi F., Ragni G. (2007). In vitro development of human oocytes after parthenogenetic activation or intracytoplasmic sperm injection. *Fertility and Sterility*.

[21] Grupen C. G., Mau J. C., McIlfatrick S. M., Maddocks S., Nottle M. B. (2002). Effect of 6-dimethylaminopurine on electrically activated in vitro matured porcine oocytes. *Molecular Reproduction and Development*.

[22] Liu L., Yang X. (1999). Interplay of maturation-promoting factor and mitogen-activated protein kinase inactivation during metaphase-to-interphase transition of activated bovine oocytes. *Biology of Reproduction*.

[23] Nixon V. L., Levasseur M., McDougall A., Jones K. T. (2002). Ca^2+^oscillations promote APC/C-dependent cyclin B1 degradation during metaphase arrest and completion of meiosis in fertilizing mouse eggs. *Current Biology*.

[24] Mead E. J., Maguire J. J., Kuc R. E., Davenport A. P. (2007). Kisspeptins: a multifunctional peptide system with a role in reproduction, cancer and the cardiovascular system. *British Journal of Pharmacology*.

[25] Wahab F., Atika B., Shahab M., Behr R. (2016). Kisspeptin signalling in the physiology and pathophysiology of the urogenital system. *Nature Reviews Urology*.

[26] Caraty A., Smith J. T., Lomet D. (2007). Kisspeptin synchronizes preovulatory surges in cyclical ewes and causes ovulation in seasonally acyclic ewes. *Endocrinology*.

[27] Castellano J. M., Gaytan M., Roa J. (2006). Expression of KiSS-1 in rat ovary: putative local regulator of ovulation?. *Endocrinology*.

[28] Gaytán F., Gaytán M., Castellano J. M. (2009). KiSS-1 in the mammalian ovary: distribution of kisspeptin in human and marmoset and alterations in KiSS-1 mRNA levels in a rat model of ovulatory dysfunction. *American Journal of Physiology-Endocrinology and Metabolism*.

[29] Jayasena C. N., Abbara A., Comninos A. N. (2014). Kisspeptin-54 triggers egg maturation in women undergoing in vitro fertilization. *The Journal of Clinical Investigation*.

[30] Matsui H., Takatsu Y., Kumano S., Matsumoto H., Ohtaki T. (2004). Peripheral administration of metastin induces marked gonadotropin release and ovulation in the rat. *Biochemical and Biophysical Research Communications*.

[31] Saadeldin I. M., Koo O. J., Kang J. T. (2012). Paradoxical effects of kisspeptin: it enhances oocyte in vitro maturation but has an adverse impact on hatched blastocysts during in vitro culture. *Reproduction, Fertility and Development*.

[32] Babwah A. V., Pampillo M., Min L., Kaiser U. B., Bhattacharya M. (2012). Single-cell analyses reveal that KISS1R-expressing cells undergo sustained kisspeptin-induced signaling that is dependent upon an influx of extracellular CA^2+^. *Endocrinology*.

[33] Constantin S., Caligioni C. S., Stojilkovic S., Wray S. (2009). Kisspeptin-10 facilitates a plasma membrane-driven calcium oscillator in gonadotropin-releasing hormone-1 neurons. *Endocrinology*.

[34] Hsu M.-C., Wang J.-Y., Lee Y.-J., Jong D.-S., Tsui K.-H., Chiu C.-H. (2014). Kisspeptin modulates fertilization capacity of mouse spermatozoa. *Reproduction*.

[35] Jiang Q., He M., Ko W. K. W., Wong A. O. L. (2014). Kisspeptin induction of somatolactin-*α* release in goldfish pituitary cells: functional role of cAMP/PKA-, PLC/PKC-, and Ca^2+^/calmodulin-dependent cascades. *American Journal of Physiology-Endocrinology and Metabolism*.

[36] Liu X., Lee K., Herbison A. E. (2008). Kisspeptin excites gonadotropin-releasing hormone neurons through a phospholipase C/calcium-dependent pathway regulating multiple ion channels. *Endocrinology*.

[37] Min L., Soltis K., Reis A. C. S. (2014). Dynamic kisspeptin receptor trafficking modulates kisspeptin-mediated calcium signaling. *Molecular Endocrinology*.

[38] Pinto F. M., Cejudo-Román A., Ravina C. G. (2012). Characterization of the kisspeptin system in human spermatozoa. *International Journal of Andrology*.

[39] Kuijk E. W., Du Puy L., Van Tol H. T. A., Haagsman H. P., Colenbrander B., Roelen B. A. J. (2007). Validation of reference genes for quantitative RT-PCR studies in porcine oocytes and preimplantation embryos. *BMC Developmental Biology*.

[40] Livak K. J., Schmittgen T. D. (2001). Analysis of relative gene expression data using real-time quantitative PCR and the 2^−ΔΔ*C*_T_^ method. *Methods*.

[41] Koo D.-B., Chae J.-I., Kim J.-S. (2005). Inactivation of MPF and MAP kinase by single electrical stimulus for parthenogenetic development of porcine oocytes. *Molecular Reproduction and Development*.

[42] Miao Y.-L., Williams C. J. (2012). Calcium signaling in mammalian egg activation and embryo development: the influence of subcellular localization. *Molecular Reproduction and Development*.

[43] Ducibella T., Huneau D., Angelichio E. (2002). Egg-to-embryo transition is driven by differential responses to Ca^2+^ oscillation number. *Developmental Biology*.

[44] Knott J. G., Gardner A. J., Madgwick S., Jones K. T., Williams C. J., Schultz R. M. (2006). Calmodulin-dependent protein kinase II triggers mouse egg activation and embryo development in the absence of Ca^2+^ oscillations. *Developmental Biology*.

[45] Im G.-S., Samuel M., Lai L., Hao Y., Prather R. S. (2007). Development and calcium level changes in pre-implantation porcine nuclear transfer embryos activated with 6-DMAP after fusion. *Molecular Reproduction and Development*.

[46] Leal C. L. V., Liu L. (1998). Differential effects of kinase inhibitor and electrical stimulus on activation and histone H1 kinase activity in pig oocytes. *Animal Reproduction Science*.

[47] Hagen D. R., Prather R. S., First N. L. (1991). Response of porcine oocytes to electrical and chemical activation during maturation in vitro. *Molecular Reproduction and Development*.

[48] Alberio R., Zakhartchenko V., Motlik J., Wolf E. (2001). Mammalian oocyte activation: lessons from the sperm and implications for nuclear transfer. *The International Journal of Developmental Biology*.

[49] Prather R. S. (2001). Basic mechanisms of fertilization and parthenogenesis in pigs. *Reprod Supp*.

[50] Fissore R. A., Robl J. M. (1992). Intracellular CA^2+^ response of rabbit oocytes to electrical stimulation. *Molecular Reproduction and Development*.

[51] Rickords L. F., White K. L. (1992). Electrofusion‐induced intracellular Ca^2+^ flux and its effect on murine oocyte activation. *Molecular Reproduction and Development*.

[52] Loi P., Ledda S., Fulka J., Cappai P., Moor R. M. (1998). Development of parthenogenetic and cloned ovine embryos: effect of activation protocols. *Biology of Reproduction*.

[53] Ruddock N. T., Machaty Z., Milanick M., Prather R. S. (2000). Mechanism of intracellular pH increase during parthenogenetic activation of in vitro matured porcine oocytes. *Biology of Reproduction*.

[54] Susko-Parrish J. L., Leibfried-Rutledge M. L., Northey D. L., Schutzkus V., First N. L. (1994). Inhibition of protein kinases after an induced calcium transient causes transition of bovine oocytes to embryonic cycles without meiotic completion. *Developmental Biology*.

[55] Whitaker M. J., Steinhardt R. A. (1982). Ionic regulation of egg activation. *Quarterly Reviews of Biophysics*.

[56] Sun L., Machaca K. (2004). Ca^2+^_cyt_ negatively regulates the initiation of oocyte maturation. *The Journal of Cell Biology*.

[57] De La Fuente R., King W. A. (1998). Developmental consequences of karyokinesis without cytokinesis during the first mitotic cell cycle of bovine parthenotes. *Biology of Reproduction*.

[58] Rajagopalan L. E., Malter J. S. (1996). Turnover and translation of in vitro synthesized messenger RNAs in transfected, normal cells. *The Journal of Biological Chemistry*.

[59] Wisdom R., Lee W. (1991). The protein-coding region of c-myc mRNA contains a sequence that specifies rapid mRNA turnover and induction by protein synthesis inhibitors. *Genes & Development*.

[60] Wodnar-Filipowicz A., Moroni C. (1990). Regulation of interleukin 3 mRNA expression in mast cells occurs at the posttranscriptional level and is mediated by calcium ions.. *Proceedings of the National Acadamy of Sciences of the United States of America*.

[61] Zhang D., Li X., Sun S., Shen X., Cui X., Kim N. (2010). Involvement of ER-calreticulin-Ca^2+^ signaling in the regulation of porcine oocyte meiotic maturation and maternal gene expression. *Molecular Reproduction and Development*.

[62] Lorca T., Cruzalegui F. H., Fesquet D. (1993). Calmodulin-dependent protein kinase II mediates inactivation of MPF and CSF upon fertilization of xenopus eggs. *Nature*.

[63] Lazar S., Galiani D., Dekel N. (2002). cAMP-dependent PKA negatively regulates polyadenylation of c-mos mRNA in rat oocytes. *Molecular Endocrinology*.

[64] Hashimoto N., Watanabe N., Furuta Y. (1994). Erratum: Parthenogenetic activation of oocytes in c-mos-deficient mice. *Nature*.

[65] Verlhac M.-H., Kubiak J. Z., Weber M. (1996). Mos is required for MAP kinase activation and is involved in microtubule organization during meiotic maturation in the mouse. *Development*.

[66] Yamamoto T. M., Iwabuchi M., Ohsumi K., Kishimoto T. (2005). APC/C-Cdc20-mediated degradation of cyclin B participates in CSF arrest in unfertilized Xenopus eggs. *Developmental Biology*.

